# Co-Amorphous Solid Dispersions for Solubility and Absorption Improvement of Drugs: Composition, Preparation, Characterization and Formulations for Oral Delivery

**DOI:** 10.3390/pharmaceutics10030098

**Published:** 2018-07-19

**Authors:** Anna Karagianni, Kyriakos Kachrimanis, Ioannis Nikolakakis

**Affiliations:** Department of Pharmaceutical Technology, School of Pharmacy, Faculty of Health Sciences, Aristotle University of Thessaloniki, 54124 Thessaloniki, Greece; karagiak@pharm.auth.gr (A.K.); kgk@pharm.auth.gr (K.K.)

**Keywords:** co-amorphous solid dispersions, drug-drug co-amorphous, drug-carboxylic acids, drug-amino acids, dissolution, absorption

## Abstract

The amorphous solid state offers an improved apparent solubility and dissolution rate. However, due to thermodynamic instability and recrystallization tendencies during processing, storage and dissolution, their potential application is limited. For this reason, the production of amorphous drugs with adequate stability remains a major challenge and formulation strategies based on solid molecular dispersions are being exploited. Co-amorphous systems are a new formulation approach where the amorphous drug is stabilized through strong intermolecular interactions by a low molecular co-former. This review covers several topics applicable to co-amorphous drug delivery systems. In particular, it describes recent advances in the co-amorphous composition, preparation and solid-state characterization, as well as improvements of dissolution performance and absorption are detailed. Examples of drug-drug, drug-carboxylic acid and drug-amino acid co-amorphous dispersions interacting via hydrogen bonding, π−π interactions and ionic forces, are presented together with corresponding final dosage forms.

## 1. Introduction

About 40% of the marketed and 90% of the drugs under development present low water solubility, resulting in poor and variable oral absorption, low bioavailability and unsatisfactory therapeutic efficacy [1,2]. As a rule, for stability reasons, the low solubility crystalline form of a drug is used in formulations. Therefore, different approaches have been explored such as salt formation, solid dispersions and amorphous homologues among others [3,4]. The amorphous solid state offers improved apparent solubility and dissolution rate due to the lower energy barrier required to dissolve the molecules, and hence transformation of crystalline drug into amorphous is widely employed for increasing solubility. However, thermodynamic instability due to the recrystallization tendency during processing, storage and in contact with the biological fluids, limits the potential application of amorphous systems [5]. Therefore, new formulation strategies based on solid molecular dispersions are being exploited and co-amorphous dispersions is one of them with very promising results [6].

## 2. Solid Dispersions—Glass Solutions

**Figure 1:**
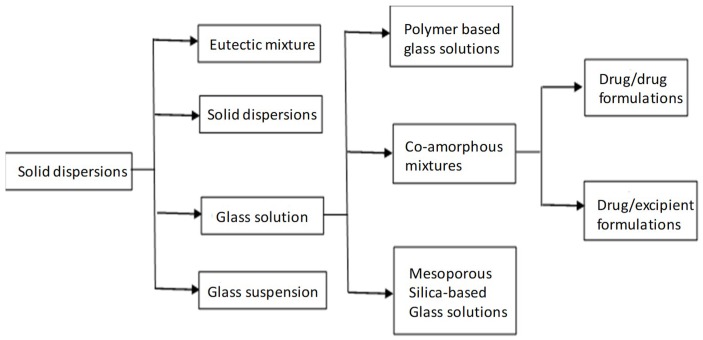
Classification chart of solid dispersions [10].

Solid dispersion (SD) was defined as the “dispersion of one or more active substances in an inert carrier prepared by melting, dissolution or melting-dissolution” [7]. They can be classified according to the solid phase and physical state as eutectic mixtures, solid solutions, glass solutions and glass suspensions (Figure 1). Glass solutions are single-phase amorphous systems. They are considered as supersaturated drug delivery systems, capable of maintaining elevated supersaturation drug levels in the gastrointestinal fluids thus, increasing absorption rate and bioavailability [8]. Amorphous SDs are further divided into polymeric and non-polymeric according to the stabilizers used [9].

In the polymeric SDs, the drug is incorporated as molecular dispersion in a glass polymeric matrix, stabilized by physical separation of the molecules inside the polymer chains. Most polymeric carriers have high glass transition temperature (Tg), and thus increase the Tg of the amorphous drug. Polymers act as stabilizers by decreasing molecular mobility, and hence inhibit nucleation and crystal growth, while molecular drug-polymer interactions can further inhibit recrystallization [11]. Nonetheless, there are problems related to polymeric SDs such as low miscibility, necessitating large polymer/drug ratios which in the case of high dosing lead to oversize dosage units. Furthermore, sensitivity to heat and humidity due to the hygroscopicity of the polymers can be an issue, since the moisture reduces the Tg with possible phase separation and recrystallization [12]. Also, since relaxation of amorphous material and recrystallization is possible below the Tg, storage at low temperatures 50 K less than Tg has been suggested [13]. Other challenges of solid drug-polymer glass solutions have poor processing ability due to their sticky nature [14]. Hence, due to possible stability problems and formulation difficulties, only a few polymeric ASDs have reached the market (KALETRA^®^, ZELBORAF^®^).

## 3. Co-Amorphous Dispersions

**Figure 2:**
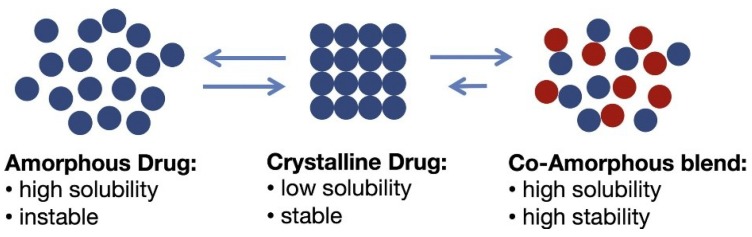
Advantages of co-amorphous dispersions over the corresponding amorphous and crystalline drugs [10].

In the last years a new strategy, alternative to amorphous polymeric, is co-amorphous solid dispersions with solubility and stability improvements over the corresponding amorphous and crystalline drugs as explained in Figure 2. They combine two or more low molecular weight (MW) ingredients into a homogenous amorphous single-phase and have been used to stabilize amorphous forms of low solubility drugs [15,16]. Due to the low MW components, low amount of stabilizer (co-former) is required, and thus oversized dose units and hygroscopicity problems inherent to polymeric amorphous SDs are avoided [10].

**Figure 3:**
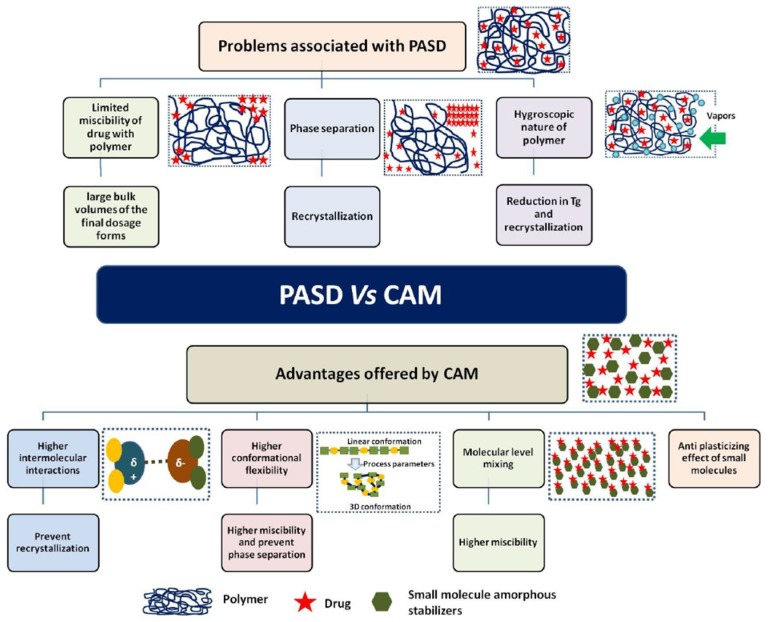
Advantages of co-amorphous systems (CAM) over polymeric amorphous solid dispersions (PASD) [23].

**Figure 4:**
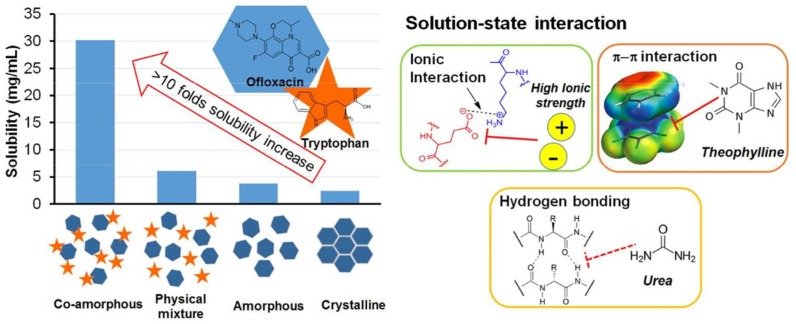
Increased solubility of the co-amorphous systems due to intermolecular interactions [22].

The advantages of co-amorphous over polymeric dispersions are presented in detail in Figure 3 and Figure 4. Co-amorphous SDs provide high drug solubility due to the high energy of the amorphous state and because no energy is needed for the rearrangement of the crystal lattice during dissolution (Figure 4). Additionally, they may exhibit high stability and improved dissolution not only compared to their crystalline homologues but also to the individual amorphous forms [17,18,19]. Significant increase of Cmax (1.3 to 30 times) and area under the curve (AUC) (1 to 5 times), as well as a decrease of Tmax have been achieved by many co-amorphous systems [20]. The main reason for these improvements is the strong solid-state interactions between the components [21]). In addition, the stability of co-amorphous mixtures is due to the increase of Tg and to the homogeneous molecular-level dispersions achieved by high energy mixing [19,20]. Impregnation with small molecules, e.g., amino acids, are considered critical for preventing recrystallization. In the majority of the studies the physical stability of such systems is attributed to intermolecular interactions such as hydrogen bonds, π−π, or even ionic interactions [22].

Co-amorphous dispersions can lead to increased stability even in the absence of interactions due to molecular mixing. In the simvastatin/glipizide system co-amorphous SD improvement in stability and solubility were observed after ball milling or cryomilling, although no detectable intermolecular interactions existed [24]. The best mixtures remained stable for over 2 months due to a stabilization mechanism similar to drug-polymer systems, whereby the amorphous component of the mixture acts as an anti-plasticizing stabilizer, which in this case was glipizide.

### 3.1. Recrystallization in Co-Amorphous Dispersions

In addition to dissolution improvement, co-amorphization helps to prevent recrystallization during storage or upon contact with biological fluids, through molecular interactions. Schantz et al. [25] studied citric acid—paracetamol as crystalline physical mixtures and as amorphous blends, using solid-state NMR. Crystallization caused phase separation and the properties of the two phases resembled those of the pure components. Pajula et al. [26] described a crystallization pathway for an amorphous, molecularly mixed binary system. According to their theory, recrystallization results from slow shearing and phase separation, nucleation and crystal growth. Recrystallization from a supersaturated solution during dissolution is also a possibility following a complex process involving breakage of intermolecular bonds and molecular rearrangement to form crystals of the individual components.

### 3.2. Co-Amorphous vs. Co-Crystals

**Figure 5:**
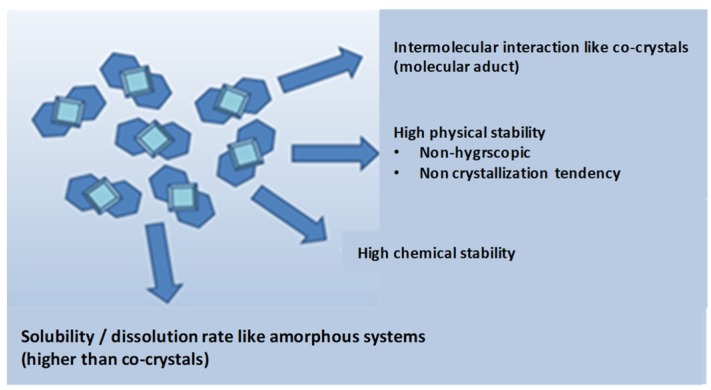
Comparison between co-amorphous and co-crystal solid dispersions.

In theory, co-amorphous SDs combine the advantages of co-crystals (molecular level mixing with high solubility component) and amorphous form (disordered arrangement) to give dissolution improvement. Comparison between co-amorphous and co-crystal dispersions is presented in Figure 5. Their common characteristic is formation in stoichiometric proportions and that can be stabilized by H-bonds. Some compounds e.g., itraconazole (ITZ) with l-tartaric acid (TA), coexist better as co-crystals, whereas others e.g., ITZ with fumaric acid (FA) as co-amorphous dispersions [27]. Until recently, only recrystallization of co-amorphous to the individual components has been reported after storage, but no recrystallization to co-crystal [28]. However, moisture is known to produce co-crystals [29] as an intermediate step prior to amorphous state [30]. 

### 3.3. Classification of Co-Amorphous Solid Dispersions

Until now, about 50 different combinations of drug-drug and drug-excipient co-amorphous systems in different molecular proportions have been reported [31].

#### 3.3.1. Drug-Drug Compositions

In these systems, two pharmacologically-related drugs are stabilized. The simultaneous administration of the drugs in one dosage unit results in better patient compliance and minimal excipients. Additionally, both drugs gain improved solubility and dissolution rate, with synchronized release. For instance, tests with the indomethacin/naproxen 1:1 co-amorphous SD demonstrated similar dissolution profiles for the two drugs with simultaneous release and also increased stability compared to the crystalline or amorphous IND, which was attributed to formation of heterodimers [19].

Yamamura et al. [32,33,34] suggested cimetidine as a carrier that forms dimeric co-amorphous mixtures with the acidic non-steroidal anti-inflammatory drugs (NSAID) naproxen, indomethacin, and diflunisal prepared by the solvent evaporation method, due to salt formation between the imidazole cimetidine ring and the carboxyl groups of the NSAIDs. Examples of investigated co-amorphous systems were: naproxen-cimetidine and indomethacin-ranitidine hydrochloride for relief of pain and of the gastrointestinal side effects of NSAIDs [15,17], simvastatin-glipizide for hypercholesterolemia and diabetes in metabolic disorders [24] and tranilast-diphenhydramine hydrochloride as anti-allergic/anti-inflammatory treatment [35]. Other studies showed that co-amorphous SDs increased the in vivo supersaturation and dissolution rate compared to individual amorphous or crystalline drugs but to a lesser extent than that expected from in vitro tests. However, supersaturation depends on dissolution factors such as the pH of the media and possible ionization, and the solubilizing effect of bile salts among others [6,36].

**Figure 6:**
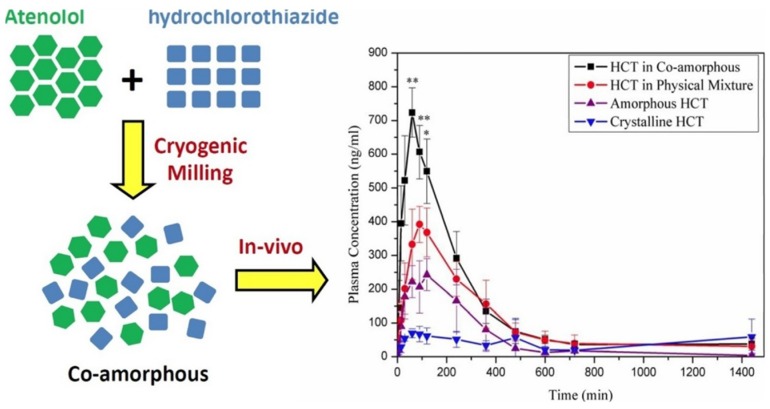
Improved concentration in the plasma of the co-amorphous hydrochlorothiazide (HCT) [38].

So far, only few studies have reported in vivo evaluation of drug-drug co-amorphous SDs [10,37]. High curcumin concentration administered as co-amorphous with artemisin was found in the plasma of male rats compared to administration alone, although it was not clear whether the increase was due to amorphization of curcumin or to co-amorphization [20]. Additionally, studies in mice showed great improvement in the bioavailability of hydrochlorothiazide co-amorphous mixture with atenolol following the order of enhancement: co-amorphous dispersion > natural mixture > amorphous drug > crystalline drug as demonstrated in Figure 6 [38].

Practically, co-amorphous drug-drug SDs have limited application, as it is difficult to combine pharmacologically related drugs that are also able to form glass solution in the required doses. Thus, the idea was extended to combinations of drugs with inert molecules such as amino acids, carboxylic acids (citrate, tartrate) weak bases (meglumine, flavonoids), saccharin or nicotinamide that are capable of hydrogen bonding and have low toxicity [39,40].

#### 3.3.2. Drug-Amino Acid Co-Amorphous Compositions and Co-Amorphous Salts

In these systems, amino acids (AAs) are used as biocompatible co-formers and act as anti-plasticizers by increasing the Tg, while blocking drug-drug interactions, delaying recrystallization. Due to the low MW, only small amounts are required compared with polymeric SDs [19,41]. Selection of AAs is based on knowledge of possible drug-receptor interactions at biological binding sites [41,42]. Improved dissolution rate and physical stability of co-amorphous drug-AA systems compared to the amorphous drug have been reported, due to molecular interaction [17,21,41,42]. However, stable systems with non-interacting components are also possible [24].

**Figure 7:**
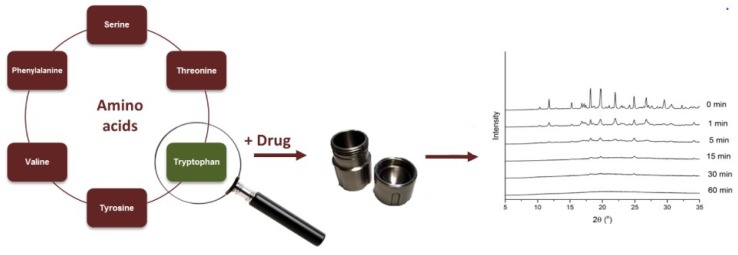
Selection of amino acids as appropriate co-formers in milling [43].

For quick screening and suitability of A.A. co-formers, Kasten et al. [43] examined the degree of co-amorphization following different milling times of six model drugs combined with 20 AAs in equimolar ratios. Selection of AA was based on >90% crystallinity reduction after 15 min milling as shown in Figure 7. The results indicated that non-polar AA such as tryptophan, phenylalanine, leucine, isoleucine, methionine, valine and proline, are first-choice co-formers. Basic AAs appear to be suitable for amorphous salt formation with acidic drugs whereas acidic AAs are generally poor co-formers.

**Figure 8:**
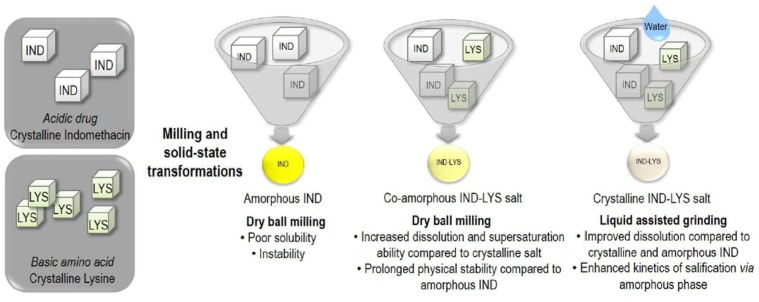
Different milling methods lead to amino acid salts with different properties [48].

Higher stability has been found in mixtures where AAs form salts [44]. Salts lead to increased Tg of the co-amorphous SD due to strong ionic interactions that resist crystallization and exert anti-plasticizing effect [45]. Furthermore, salt formation enhances dissolution because the dissolved ions make ion-dipole interactions with water molecules, which is energetically more favorable than hydrogen bonding between water and a non-ionized drug. However, amorphous salt formation is limited to pKa differences between components greater than 3, and hence they are applicable to a limited number of drugs. Figure 8 shows the result of the application of different milling methods. It can be seen that different methods result in products with different characteristics.

### 3.4. Selection of Amino Acid Co-Formers

The fact that certain AAs can be co-amorphisized successfully with one drug but not with another led to the need for systematic studies for co-former selection, using computational methods and theoretical approaches. Detection of intermolecular interactions with FTIR, Raman and NIR spectra is a good indication for the formation of stable co-amorphous SDs [18,46]. Spectroscopic data can be aided by quantum mechanics [31]. Using density functional theory calculations (DFT), quantum molecular theory (QTAIM) and natural bond orbital analysis (NBO), Russo et al. [47] confirmed the formation of omeprazole-amoxicillin heterodimer.

Ueda et al. [49] used multiple variable analyses to study the effect of physicochemical properties of co-formers. They underlined the importance of crystallization tendency (Tg/melting temperature (Tm) ratio), molecular flexibility, reduced glass transition (Tg/Tm), number of hydrogen bond acceptors, topological polar surface area and polarizability in the formation of stable co-amorphous SDs. The importance of molecular size and flexibility was demonstrated by the suitability of the flexible tryptophan but not of the rigid proline and the high tendency of the last to be organised in a crystal lattice. Alhalaweh et al. [50] developed a predictive computational tool based on two molecular markers, the number of hydrogen bond acceptors and the Huckel pi atomic charges that helped the screening of 77 out of 131 drug molecules that formed glass dispersions. Cimetidine, indapamide, quercetin and ritonavir were found to be potent glass-forming agents, capable of amorphous state stabilization. Further studies by Pajula et al. [51] showed that miscibility in the co-amorphous SD is the most important factor, while Tg increase, molecular mixing, interactions and stabilization follow in terms of importance. Partial or non-miscibility can dramatically influence the recrystallization tendency of a stabilized co-amorphous drug after phase separation.

Jensen et al. [52] studied the ease of amorphization and mechanism of coamorphization during ball milling of two coamorphous drug−amino acid systems, indomethacin−tryptophan (Ind−Trp) and furosemide−tryptophan (Fur−Trp), at a 1:1 molar ratio toward their ease of amorphization and mechanism of coamorphization during ball milling. Their physicochemical properties were investigated using XRPD, 13C solid state NMR (ssNMR), and DSC. Comilling the drug with the amino acid reduced the milling time required to obtain an amorphous powder. Amorphization was observed as reductions in XRPD reflections and was additionally quantified based on normalized principal component analysis (PCA) scores of the ssNMR spectra. Furthermore, the evolution in the Tg of the coamorphous systems over time indicated complete co-amorphization after 30 min of milling. From the DSC data the formation mechanism of the coamorphous systems was elucidated. The Tgs suggested that coamorphous Ind−Trp was formed by the amino acid being dissolved in the amorphous drug, whereas coamorphous Fur−Trp was formed by the drug being dissolved in the amorphous amino acid.

For prediction of the components miscibility, the Flory-Huggins interaction parameter (χ) [51] and the Hansen solubility parameter (δ) [41,52] have been employed. For thermally stable compounds, miscibility of components in the melted state can be easily determined from a phase diagram. Marsac et al. [53] used a melting point depression approach, whereby miscible systems showed mp depression of the drug, while immiscible or partially miscible systems showed little or no depression. More research using larger data sets and advanced statistical tools is necessary in order to develop systematic approaches for the selection of co-formers [31].

### 3.5. Dissolution Rate from Co-Amorphous Solid Dispersions

**Figure 9:**
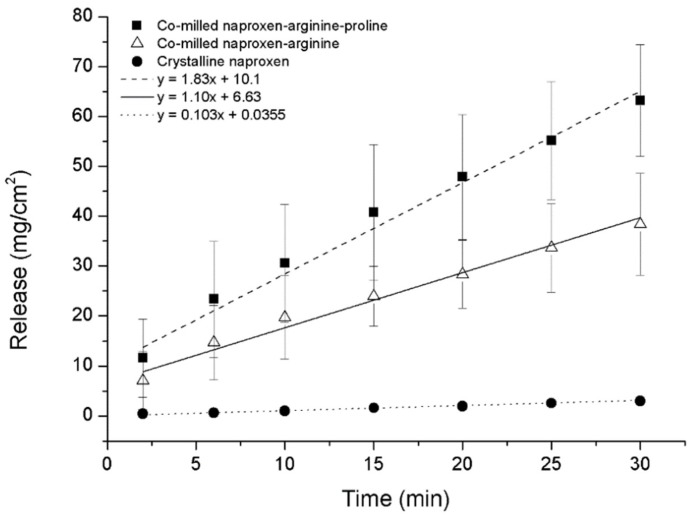
Improved dissolution rate by adding a high-water solubility amino acid in ternary mixtures [46].

Dissolution rate from the co-amorphous system depends on co-former solubility and on the strength of the drug-co-former interaction. In a study of ternary co-amorphous mixtures of naproxen-tryptophan-proline, and naproxen-arginine/proline, naproxen was stabilized by co-amorphization with the high Tg tryptophan or via salt formation with arginine [46]. The addition of PRO in NAP-TRP and NAR-ARG binary mixtures contributed to further hydrogen bonding and stabilization by enhancing interactions and increasing the dissolution rate from the ternary mixtures. An increased intrinsic dissolution rate (IDR) was also found for the NAP-ARG co-amorphous salt, and even higher IDR for the ternary NAP-ARG-PRO due to the addition of the high-water solubility amino acid as shown in Figure 9. However, a later study of the co-amorphous indomethacin-lysine (IND-LYS) SD [48] showed that the highly soluble LYS facilitated dissolution only to a certain extent. Since the solubility of LYS is higher than the drug and interactions between the two are not strong, the highly soluble co-former dissolved much faster leaving a recrystallized drug.

### 3.6. Molecular Proportion in Co-Amorphous Solid Dispersions

Molecular proportion plays a significant role in stability and 1:1 molar ratios usually indicate optimal physical stability due to the large number of specific intermolecular interactions by formation of heterodimers through H-bonds or ion coupling [17,19]. The increased physical stability is attributed to the complexity of the recrystallization process, which involves the breaking of the heterodimer intermolecular bonds and formation of homodimers. Because the strong molecular interactions remain in contact with water, recrystallization during dissolution is prevented [54]. For other molar ratios, it has been observed that the co-amorphous SDs recrystallize to pure components, with the excess component crystallizing faster [15,19], unless other mechanisms are involved [24]. However, there have also been studies [35,55] showing stability for other molecular proportions. For example, 2:1, 1:1 and 1:2 compositions of tranilast-diphenhydramine systems remained amorphous during 30 days and 1: 1 and 1:2 co-amorphous dispersions of IBU-ARG and IND-ARG did not crystallize, probably due to hydrogen bonding along with salt formation, which can explain the high stability of the 1:2 composition. An interesting case is the co-amorphous NAP-IND mixtures, in which the 60:40 molar ratio was found to be a eutectic and hence the most stable [56].

## 4. Solid State Characterization

Solid state characterization is important for verification of co-amorphous formation and in stability studies, for the initiation of crystallization. X-Ray Powder Diffraction (XRPD), Differential Scanning Calorimetry (DSC), Fourier-Transform Infrared Spectroscopy (FTIR) and emerging variations of these methods have been the basis for characterizing co-amorphous systems.

### 4.1. XRPD and DSC

X-Ray Powder Diffraction (XRPD) provides information on the presence of crystalline phase and indirectly on amorphicity as manifested by the presence of reflections and halos, respectively, and is always applied in the study of amorphous systems. These are single-phase homogeneous systems, reflected in the DSC thermograms by a single Tg corresponding to the temperature of transformation of the amorphous material from its glass state to a subcooled or viscous fluid. The single Tg indicates miscibility, i.e., one of the co-amorphous components acts as a crystallization inhibitor for the other [21]. Modulated Differential Scanning Calorimetry (mDSC) has also been used, as it solves the problem of overlapping thermal transitions [55,57,58,59]. The Tg of a binary co-amorphous SD tends to be closer to the excess component and is usually increased compared to the individual amorphous components, indicating stability and a reduced crystallization tendency [5,17]. However, there have been cases where co-amorphous SD with higher Tg was not the most stable, showing that Tg is not the only factor for the stability of co-amorphous systems [54].

**Figure 10:**
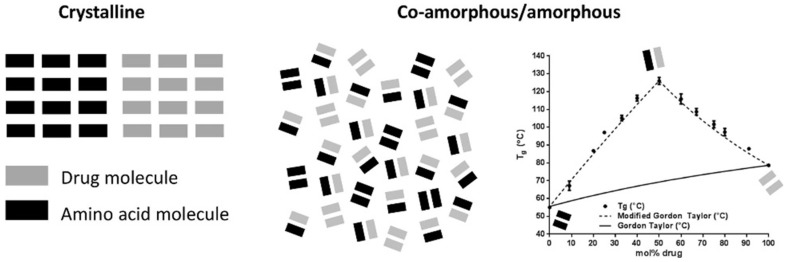
Glass transition point of a co-amorphous system in comparison to the theoretical from the Gordon-Taylor equation [54].

The Tg of mixtures is estimated from the Gordon-Taylor equation which considers ideal miscibility without interactions. Negative deviation of experimental Tg is ascribed to overall loss of bonds during mixing and to the residual water acting as a plasticizer, decreasing Tg or increasing the free volume. Positive deviations indicate that the number and strength of bonds between the co-amorphous components are stronger than inside the individual components [60]. This is exemplified in Figure 10. In most cases, Tg is assessed after preparation of the co-amorphous SD, although significant increases up to 15 °C have been observed due to structure relaxation [61].

### 4.2. Fourier-Transform Infrared Spectroscopy—FTIR

FTIR is valuable for detecting changes in the solid state related to the molecular arrangement of co-amorphous SDs [62]. It detects changes in the solid state such as hydrogen bonding or π−π interactions which are reflected as peak shifts of functional groups [63]. The IR spectrum of the co-amorphous SD is compared with that of the individual components in the amorphous state, which for some amino acids may be a difficult task. In general, the peaks in the co-amorphous spectra are broader, lower in intensity and shifted compared to the component amorphous or crystalline spectra, indicating modification of molecular structure [64,65]. The less well-defined peaks in the co-amorphous spectra as compared to the components is an indication of greater disorder, even in the case of not interacting components mixed at molecular level [24].

Significant peak shifts during the transformation of drugs to amorphous occur between 1000 and 1800 cm^−1^, providing information on aromatic systems (1100–1500 cm^−1^), carboxylic acids (1700 cm^−1^) and amides (1600 cm^−1^), and at 3100–3700 cm^−1^ due to –OH vibrations. Small differences in the molecular arrangement and/or environment due to non-specific intermolecular interactions, H-bonds or π−π interactions underline interactions and formation of heterodimers [12,66]. Changes in the FTIR spectra may also indicate salt formation e.g., between IND and ARG in co-amorphous SDs or even esterification e.g., between venlafaxine and citric acid [55,67].

### 4.3. Newer Techniques

Although XRPD, DSC and FTIR have been the most widely used techniques for characterizing co-amorphous solid dispersions, newer techniques have emerged which can provide further information at higher speeds. Fast scanning differential scanning calorimetery (DSC) can be applied to overcome kinetically controlled and temperature-dependent thermal events due to the rapid scanning rates which is of particular advantage for the analysis of amorphous systems, whose properties may change during scanning due to enthalpy relaxation or crystallization [68]. Microthermal analysis that combines various techniques including scanning thermal microscopy, localized thermomechanical and differential thermal analysis, nanothermal analysis and thermally assisted particle manipulation has been studied. It allows fast localized heating, thus effecting the analysis (e.g., Tg, recrystallization, melting) without compromising the integrity of the entire sample.

Further spectroscopic techniques that have been used to analyze intermolecular interactions in co-amorphous SDs and to quantify crystallinity are Raman, NIR and SS-NMR [17,25,68,69]. Scanning electron microscopy (SEM) has also been used to demonstrate amorphous aggregates and the absence of crystalline forms that show characteristic geometry [69,70,71]. Nonlinear optical (NLO) techniques such as second harmonic generation, coherent antistokes Raman scattering, stimulated Raman scattering, and two-photon fluorescence have also been implemented for the solid-state characterization of pharmaceutical materials [72]. These combined techniques offer several advantages such as speed, selectivity, quantitation and, therefore, could be particularly useful as they could facilitate formulation development and the testing required to bring new, higher quality drugs to the market. For a more detailed account of microscopic, surface analysis, thermal analysis and spectroscopic analytical methods, the reader is referred to specialized review articles [68,73].

In general, a single method is not enough for characterization. Therefore, a combination of established and newer techniques is employed to elucidate the mechanisms of destabilization and the development and viability of co-amorphous dispersions. In the following paragraph, information on previous results gained on co-amorphous formulations are presented.

### 4.4. Studies Using Combinations of Analytical Methods

➢ Chieng et al. [15] studied amorphous c-indomethacin and ranitidine hydrochloride form 2 at various weight ratios (1:2, 1:1 and 2:1). During co-milling, the XRPD characteristic peaks of indomethacin were found to decrease faster than those of ranitidine hydrochloride and both drugs were fully converted into the amorphous state after 60 min. DSC results were in agreement with XRPD and Tgs of the amorphous mixtures were in good agreement with the predicted Tgs using the Gordon–Taylor equation. DRIFTS spectra showed interaction at the carboxylic acid carbonyl (HO–C=O) and benzonyl amide (NC=O) of the indomethacin molecule with the aci-nitro (C=N) of ranitidine hydrochloride.
➢ Alleso et al. [17] studied the binary systems of naproxen and cimetidine. DSC showed that the 1:1 sample was the most physically stable even though it did not have the highest Tg when compared to the 1:2 sample. Raman spectroscopy suggested a 1:1 solid-state interaction between the imidazole ring of cimetidine and the carboxylic acid of naproxen in the amorphous sample, indicating that the stabilization of the amorphous binary system was dictated by molecular-level interactions.
➢ Lobman et al. [19] studied naproxen with γ-indomethacin. Tgs were comparable with those from the Gordon Taylor equation. XRPD results showed that naproxen could be made amorphous in combination with indomethacin while this was not possible with naproxen alone. Peak shifts in the FTIR spectra indicated molecular interactions between both drugs, and it is suggested that the two drugs formed a heterodimer.
➢ Zhu et al. [22] studied Tryptophan—ofloxacin at a 1:1 weight ratio. XRPD showed complete co-amorphization and DSC showed an elevated Tg compared with the theoretical value. FTIR revealed that hydrogen bonding and π−π stacking were possibly involved in the formation of co-amorphous system.
➢ Lobman et al. [24] studied mixtures of simvastatin (SVS) and glipizide. Formation of single phase co-amorphous mixtures with mixture ratios of 2:1, 1:1 and 1:2 was detected by DSC. The observed single, concentration-dependent Tgs were found to be lower than those predicted (Gordon–Taylor equation), indicating the absence of intermolecular interactions between the two drugs, which was verified by FTIR spectral data analysis.
➢ Yamamoto et al. [27] studied interactions of itraconazole (ITZ) with fumaric (FA) or l-tartaric acid (TA) by Raman spectroscopy. ITZ-FA co-amorphous was found physically and chemically stable, whereas the ITZ-TA co-amorphous, was physically stable but chemically unstable.
➢ Sovago et al. [28] studied the mixture sodium naproxen-lactose-terahydrate. XRPD revealed the amorphous nature of the mixtures and DSC showed that the blends were single-phase co-amorphous systems as indicated by a single Tg.
➢ Ueda et al. [35] studied tranilast (TRL) and diphenhydramine hydrochloride (DPH) system. The Tg of the co-amorphous TRL-DPH deviated from the theoretical value and the enthalpy relaxation rate of the amorphous drugs (reflecting molecular mobility) was reduced due to the formation of a co-amorphous system. The intermolecular interactions between TRL and DPH in the co-amorphous system were measured from changes in the IR spectra.
➢ Teja et al. [37] studied amorphous forms of Talinolol (TLN) using Naringin (NRG) as a stabilizer. The temperature stability of prepared amorphous forms was attributed to strong intermolecular hydrogen bonding between TLN and NRG, which was confirmed by FTIR.
➢ Moinuddin et al. [38] studied the system of atenolol (ATE) with hydrochlorothiazide (HCT). The co-amorphous ATE-HCT sample with 1:1 molar ratio showed excellent physical stability, which could be attributed to the formation of strong molecular interactions between ATE and HCT as evidenced by FT-IR spectra.
➢ Gao et al. [39] studied repaglinide (REP) with saccharin (SAC). Coamorphous REP–SAC at 1:1 stoichiometry had unique thermal behavior, distinct FTIR shifts and an absence of a sharp diffraction peak, suggesting the formation of a co-amorphous material and the interaction of REP with SAC through hydrogen bonds formed between REP’s secondary amine and SAC’s carbonyl group.
➢ Shayanfar et al. [40] studied atorvastatin (ATC) and nicotinamide (ATC-NIC) characterized using DSC, FT-IR and PXRD. Crystalline ATC was converted to a co-amorphous form due to molecular interactions between ATC and NIC.
➢ Jensen et al. [44] studied indomethacin (IND)–amino acid dispersions. The mixtures were characterized for solid-state properties by DSC, thermogravimetric analysis (TGA) and for molecular interactions by XRPD and FTIR. Elevated TGs were found for all mixtures compared with the pure amorphous drug due to co-amorphization with the amino acids. Furthermore, strong intermolecular interactions in the form of salt/partial salt formation between the drug and amino acids were seen for all blends.
➢ Russo et al. [47] studied mixtures of omeprazole–amoxicillin trihydrate (CGM samples) and omeprazole–anhydrous amoxicillin (CGMa samples) at 3:7, 1:1 and 7:3 molar ratios, respectively. These systems were characterized by DSC, FTIR, PXRD, SEM and solid state uclear Magnetic Resonance (ssNMR). The co-grinding process that was used for the preparation was not useful to get a co-amorphous system but it led to disordered phase at the 1:1 CGMa ratio. Moreover, in this system both FTIR and ssNMR analysis strongly suggested intermolecular interactions between the sulfoxide group of omeprazole and the primary amine of anhydrous amoxicillin.
➢ Kasten et al. [48] prepared different physical forms of salts of indomethacin (IND) with the amino acid lysine (LYS). XRPD, FTIR and DSC showed that dry ball milling led to the formation of a fully co-amorphous salt with a single glass transition temperature (Tg), while liquid-assisted grinding resulted in a crystalline salt while a new melting point (223 °C).
➢ Jensen et al. [54] evaluated four co-amorphous systems (Ind-Trp, Fur-Trp, Ind-Arg, and Fur-Arg) mixed at different molar ratios by determination of the deviation between the experimentally determined and the calculated Tg (Gordon-Taylor). They confirmed that the excess component was amorphous within the co-amorphous mixture. FTIR and ssNMR data did not support the presence of additional intermolecular drug-amino acid interactions.
➢ Orajinta et al. [55] studied mixtures of ibuprofen-arginine and indomethacin-arginine mixtures at two drug-arginine molar ratios (1:1 and 1:2) using DSC, XRPD and FTIR. All mixtures exhibited a single Tg, indicating the formation of homogenous single-phase systems. FTIR revealed strong interactions (mainly salt formation) that accounted for the positive deviation between measured and estimated Tg.
➢ Beyer et al. [56] studied different naproxen-indomethacin (NAP/IND) ratios prepared by melt-quenching at three cooling rates using XRPD and FTIR, both directly after preparation and during storage. All cooling methods led to amorphous samples, but with different physical stabilities.
➢ Beyer et al. [57] developed a quantification model based on XRPD and multivariate partial least squares regression approach that enabled the simultaneous determination of up to four solid state fractions: crystalline naproxen, γ-indomethacin, α-indomethacin as well as co-amorphous naproxen-indomethacin.
➢ Lobman et al. [65] studied naproxen (NAP) and indomethacin (IND) using FTIR and quantum mechanical calculations. The structures of both drugs were optimized as monomer, homodimer and heterodimer using density functional theory. Vibrational modes were computed and compared with experimentally observed spectra. It was shown that both drugs existed as homodimers in their respective individual amorphous form, but as heterodimers in the co-amorphous mixture when quench cooled together from the melt in a 1:1 molar ratio.
➢ Dengale et al. [66] studied mixtures of Ritonavir (RTV) with the known amorphous stabilizing small molecule (IND). Co-amorphous RTV-IND samples were prepared at various molar ratios (2:1, 1:1 and 1:2) and their amorphicity was confirmed by XRPD, DSC and FTIR. The Tg values were in agreement with the theoretical (Gordon–Taylor equation), which also agreed with the FT-IR results that showed no evidence of intermolecular interactions.
➢ Wairkar et al. [70] studied Nateglinide (120 mg) and Metformin hydrochloride (500 mg) mixture as a dose combination. XRPD and DSC confirmed amorphism and FTIR gave evidence of hydrogen bonding interactions (proton exchange between Nateglinide and Metformin hydrochloride).
➢ Laitinen et al. [74] studied mixtures of simvastatin and glibenclamide with the amino acids aspartic acid, lysine, serine and threonine. XRPD, DSC and FTIR revealed that the 1:1 molar combinations of simvastatin-lysine, glibenclamide-serine and glibenclamide-threonine and the 1:1:1 molar combination glibenclamide-serine-threonine formed co-amorphous mixtures.
➢ Qian et al. [75] studied lurasidone hydrochloride (LH) (pH-dependent solubility) with saccharin (SAC) at a 1:1 molar ratio. DSC and XPRD showed formation of the co-amorphous system and peak shifts in the FTIR spectra indicated the formation of a charge-assisted hydrogen bond between the NH group of LH and the C=O group of SAC.
➢ Lim et al. [76] studied indomethacin–cimetidine and naproxen–cimetidine co-amorphous systems (1:1 molar ratio) by PXRD and DSC. Structural relaxation was estimated from the Kohlrausch–Williams–Watts (KWW) relationship. The Tg was about 20 °C higher than that predicted from the Fox equation.
➢ Cray et al. [77] used sodium lauryl sulfate (SLS) as a solubilizer, adding in the co-amorphous simvastatin–lysine (SVS-LYS) 1:1 molar mixtures. Energy-dispersive X-ray spectroscopy (EDS) revealed that SLS coated the SVS and SVS-LYS particles upon spray drying. XRPD and DSC showed that in the spray-dried formulations the remaining crystallinity was due to SLS.
➢ Beyer et al. [78] optimised the physicochemical properties of co-amorphous naproxen-indomethacin dispersion by the incorporation of naproxen sodium. The influence of naproxen-sodium on the resulting physicochemical properties was analyzed by XRPD, FTIR, DSC. Fully amorphous samples with single glass transition temperatures could be prepared with naproxen molar fractions up to 0.7 which lasted for 270 days of storage.
➢ Huang et al. [79] studied combinations of valsartan (VAL) with l-histidine, l-arginine, and l-lysine with XRPD and DSC. The Tg values of the co-amorphous mixtures were higher than those of the single components and the predicted Tg values of the combinations (Gordon Taylor equation) suggesting a strong interaction between VAL and the amino acids.
➢ Pan et al. [80] studied the effect of different polymorphs of azelnidipine (AZE) on the micro-structure of the complexes with oxalic acid (OXA) using XRPD, modulated DSC and TGA, cryo-field emission, SEM, FTIR and solid-state nuclear magnetic resonance spectroscopy. AZE-OXA co-crystal was produced at a molar ratio of 2:1 when grinding with a solvent, while co-amorphous forms were obtained under dry grinding.

## 5. Preparation of Co-Amorphous Solid Dispersions

**Figure 11:**
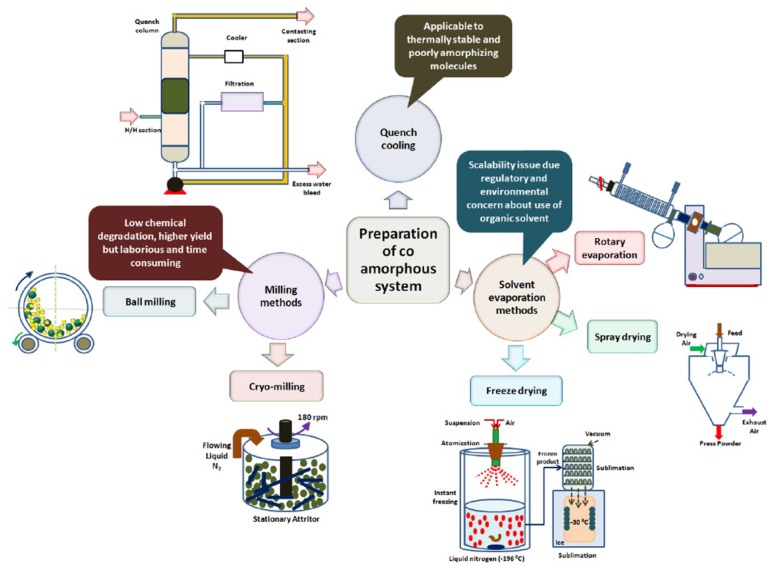
Preparation methods of co-amorphous systems [23].

The preparation techniques of amorphous systems are presented in Figure 11. Unfortunately, hot-melt-extrusion was not included but this method is described later in Section 5.6. They can be classified into melting (e.g., hot-melt extrusion), solvent (e.g., solvent evaporation, freeze drying, spray drying) and techniques involving mechanical activation (e.g., milling) [81]. Furthermore, they can be divided into thermodynamic and kinetic. The former has as starting point a thermodynamically stable non-crystalline form, i.e., the drug in melt or in solution and co-amorphous is obtained by quenching or precipitation respectively. The kinetic pathway involves direct conversion of the solid crystalline into its amorphous state. This is possible during milling due to the continuous introduction of crystalline defects and disturbances through shearing, crushing and impact, and the overall mechanical activation. Selection of the preparation method depends on the thermal stability, melting temperature and the crystallization tendency of the drug and excipients, and it is very important for the quality and stability of the amorphous product.

Co-amorphous SDs are still at an early stage of development and the majority of the studies laboratory methods such as quenching, solvent evaporation and ball milling have been applied [23]. These techniques are fast and easy, and are suitable for screening small samples.

### 5.1. Quench Cooling

The physical mixtures of the components are melted in a heated vessel under agitation and the melt is rapidly cooled on a plate over ice or liquid nitrogen. Quench cooling has the ability to quickly assess critical physicochemical parameters such as Tg, miscibility and recrystallization, as co-amorphous can be prepared and analyzed in situ in DSC sample discs. Ritonavir-indomethacin (RTV-IND) could not be formed as a co-amorphous system with the solvent evaporation technique but it was possible by quench cooling [66]. Nevertheless, quench cooling cannot always be applied (e.g., atenolol) due to thermal degradation at high temperatures [38].

### 5.2. Vibrational Ball Milling

This is the main preparation method for co-amorphous SDs. The induced mechanical activation causes distortion of the crystal lattice and by the gradual mixing of the components, leads to transitions from crystalline to amorphous state [82], co-amorphization [41,42]) and salt formation [44]. An advantage of the method is the small chemical degradation (providing the processing temperature is low) and high recovery compared to other methods [46].

However, mechanical activation can lead to incomplete disruption leaving behind crystalline regions or high energy points that may lead to recrystallization in the course of time or thermal degradation. Also, vibration ball mill can be time consuming [17,24,52], while selection of the co-former is made by trial and error. In general, the ability of mechanical activation to produce amorphous transitions depends on the vibration frequency, milling time and temperature. Milling far below Tg usually leads to amorphization, while above Tg to polymorphic transitions.

During milling and co-amorphous formation, the Tg is monitored as an index of whether the drug or the co-former is first converted, creating the amorphous phase in which the other dissolves. Jensen et al. [52] studied the co-amorphization of indomethacin (IND) and furosemide (FUR) with tryptophan (TRP). Increased Tg value of the IND-TRP dispersion compared to pure IND suggested that co-amorphous IND-TRP mixture was formed by the amino acid dissolving in the amorphous drug, whereas the decreased Tg value of the co-amorphous FUR-IND compared to pure FUR suggested that the drug dissolved in the amino acid.

**Figure 12:**
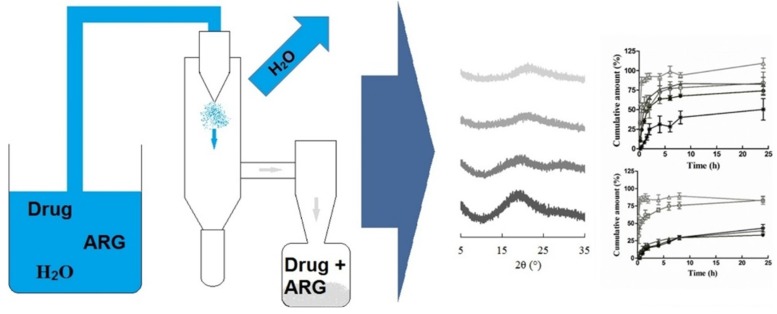
Preparation of co-amorphous systems by spray drying aqueous solution [55].

The influence of the milling process was studied in the IND-LYS co-amorphous salt dispersion by two methods: Dry ball milling (DBM) and solvent grinding (LAG), where a drop of solvent is added during milling, acting as a medium for increased molecular diffusion and to facilitate a possible reaction of the components. The results showed that dry milling led to the formation of a fully co-amorphous salt, while when water was added a crystalline synthesis was obtained, evidenced by the appearance of crystalline reflections of the salt IND-LYS after 15–60 min, attributed to a water promoting reaction after an intermediate amorphous state step. The co-amorphous composition showed high Tg and strong ionic interactions that were reflected in the increased physical stability (36 weeks) as compared to the pure amorphous drug (<7 days). Also, the dry ball milling SD showed a higher dissolution rate of 2.8, 38.6 and 90.0 times higher than that of crystalline salt (Figure 12), amorphous IND and crystalline IND [48]. However, dissolution rate was higher at the beginning of dissolution followed by a reduction, possibly due to recrystallization of amorphous IND upon contact with the aqueous dissolution medium [83].

### 5.3. Cryomilling

This is usually carried out in a vibrational ball mill. The milling bowls with components and grinding spheres are immersed in liquid nitrogen to ensure cryogenic conditions. After milling they are placed in a desiccator and allowed to reach room temperature. Milling temperature is important for solid state transitions [84]. At T < Tg, the materials become fragile and the disorganized state formation is favored by mechanical activation [85]). Cryomilling is considered more effective than conventional dry milling for producing co-amorphous SDs [24]. Laitinen et al. [74] studied co-amorphous SDs of simvastatin (SVC) and glibenclamide (GBC) with aspartic acid, lysine, serine and threonine using cryomilling. Solid-state characterization showed that 1:1 molar combinations of simvastatin-lysine, glibenclamide-serine and glibenclamide-threonine and 1:1:1 combination glibenclamide-serine-threonine formed co-amorphous SDs by weak intermolecular interactions.

### 5.4. Solvent Evaporation

This is a common method for preparing co-amorphous SDs, whereby crystalline components are dissolved in a common organic solvent which is then evaporated under vacuum or by heating. Any residues are completely removed by drying for at least 24 h [24]. Due to the rapid removal of the solvent, the molecules have no time to rearrange in a crystal lattice and they are solubilized in the co-amorphous system by co-precipitation. There have been cases however, e.g., atenolol-hydrochlorothiazide (ATE-HCT), where the method failed due to crystallization during evaporation [38]. Qian et al., 2015 [75] prepared co-amorphous lurasidone HCl-saccharin (LC-SAC) dispersion by rotary vacuum evaporation. Co-amorphous LH-SAC showed superior physical stability than amorphous LH, improved pH-independent solubility, enhanced intrinsic dissolution rate (IDR) and supersaturated dissolution.

### 5.5. Spray Drying

This is the first large-scale production method of co-amorphous drug-amino acids SDs with comparable properties to those prepared by milling [52]. It is easily applied, and able to produce stable glass solutions and solid dispersions [86]. A solution of the components is sprayed into a hot air stream where the droplets formed and dried by rapid solvent evaporation resulting in spherical, narrow size distribution co-amorphous particles [81,87]. Inlet temperature and feed rate are the most critical parameters, since they control thermal stressing and evaporation which are important in order to avoid crystallization. The product should be well-dried, since any solvent residues can reduce Tg and enhance crystallization or solvate formation [88]. Thermal exposure during spray drying should be kept below the Tg of the materials to avoid crystallization. If T outlet > Tg, the material sticks to the wall of the drying cyclone and the yield is low. For this reason, materials with low Tg cannot be processed to co-amorphous SDs by spray drying [89].

#### 5.5.1. Comparison of Spray Drying with Milling

Co-amorphous dispersions prepared by different methods showed differences in critical physicochemical properties, structural relaxation, recrystallization tendency and dissolution rate [76,90]. While spray dried samples of sodium naproxen-lactose remained co-amorphous after 5 months; co-grounded samples showed crystallinity within 7 days [28]. The disadvantage of milling compared to spray drying is demonstrated in the study of three IND-basic amino acid mixtures, IND-ARG, IND-HIS and IND-LYS by Jensen et al. [44]. While IND-ARG formed co-amorphous after 90 min milling, co-amorphization of IND-HIS and IND-LYS was not possible even after 360 min. In contrast, all three systems showed co-amorphization after spray drying solutions in acetone/water, due to strong interactions resulting in salt or partial salt formation. All mixtures were physically stable for over 10 months under dry conditions.

#### 5.5.2. Spray Drying Applications with Aqueous Solutions

Spray drying has been applied for the preparation of co-amorphous dispersions using mostly organic solutions [44,77,88]. An example of the preparation of a co-amorphous system using Arginine as co-former by spray drying aqueous solution is shown in Figure 12. However, for reasons of toxicity (solvent residues) and environmental safety, aqueous solutions using surfactants as solubilizers are preferred. To enable spray drying of aqueous drug solutions, surfactants have been used [77]. Co-amorphous drug-amino acid SDs were formed by spray drying aqueous simvastatin (SVS) solution solubilized with *sodium lauryl sulfate* (SLS). Twelve months stability was achieved with the ternary SLS-SVS-LYS combination, and the amount dissolved was two-fold higher than the corresponding physical SVS-SLS mixture and SVS-LYS co-amorphous dispersion. The stabilizing effect was attributed to partial miscibility with the drug and to the protective shell formed by SLS, separating physically and protecting the amorphous content from environmental humidity. However, tolerability of surfactants in the body is an issue and their use is not generally encouraged.

Ojarinta et al. [55] successfully prepared co-amorphous IND-ARG and IBU-ARG dispersions by spray drying aqueous solutions. High physical stability together with high Tg indicated strong interactions with salt formation, while no crystallization was observed during 1-year stability study of 1:1 or 1:2 mixtures. In addition, at low pH, co-amorphous SDs dissolved faster and more completely compared to physical drug-ARG mixtures or pure drugs. In the study by Craye et al. [77], 1:1 molar simvastatin-lysin (SYM-LYS) co-amorphous mixture was prepared by spray drying aqueous solutions using SLS as a solubilizer and 1-year stability was recorded. Therefore, spray drying offers a good alternative for the preparation of co-amorphous SDs, when other methods fail due to low yield, thermal degradation or incomplete amorphization. The difficulty is to find a common solvent for all components since poorly water-soluble drugs are combined with highly soluble co-formers.

### 5.6. Hot-Melt-Extrusion (HME)

This is a continuous solvent-free process and has been applied in the pharmaceutical industry for the production of crystalline or amorphous polymeric SDs. It is amenable to in situ monitoring for the detection of co-amorphization, degradation and intermolecular interactions by the application of Process Analytical Technology [91]. Application of HME requires consideration of the process variables (zone temperatures, speed of screw rotation or transit time and feed rate) and materials, which significantly affect the type of produced dispersion, the yield and the mechanical properties of the extrudate.

Arnfast et al. [92], added PEO (polyethylene oxide) as a plasticizer to the indomethacin-cimetidine (IND-CIM) system during HME. PEO reduced the melt viscosity, prevented phase separation (despite Tg reduction) and resulted in a ternary amorphous SD where PEO acted as co-solvent for both drugs. Bounartzi et al. [67] applied HME using the ternary mixture citric acid-Eudragit RSPO-venlafaxine hydrochloride (CA-RSPO-VEN) and produced co-amorphous transparent SD. Since in the binary CA-VEN extrudate some crystalline reflections of plasticizer existed, it was concluded that the matrix polymer was essential for the formation and stabilization of the CA-VEN co-amorphous dispersion.

HME has not been applied extensively to drug-amino acid co-amorphization, because AAs degrade above 200 °C and because the mechanical and thermal energy (short residence time) may be inadequate to overcome the crystal lattice energy and achieve amorphization. In spite of that, Lenz et al. [93] succeeded in preparing co-amorphous drug-amino acid formulations using copovidone (COP) as a matrix polymer for IND-ARG mixture. Although the co-amorphous IND-ARG-COP dispersion showed increased dissolution, phase separation was found, which was not the case when spray drying was applied to the same composition. Martínez et al. [61] proposed the use of phase diagrams based on the melting behavior of physical mixtures for the development of co-amorphous SDs with high crystalline drug content. This is because in this case the minimum HME processing temperature is controlled by the melting of crystalline solids rather than polymer relaxation.

### 5.7. Other Methods

Inkjet printing (dimethylsulfoxide, propylene glycol and water) was used by Wickstrom et al. [94] for the preparation of indomethacin-arginine (IND-ARG) co-amorphous SD. The produced formulations showed increased dissolution rate and the method could be used for the production of personalized drug delivery systems, provided that hurdles associated with the printer, ink and substrate can be overcome. Furthermore, co-amorphization of IND with ARG has been achieved, although with low yield, by freeze-drying from aqueous solutions [95]. An interesting finding was the immediate transition of a hydrated NaN-Lact-4H_2_O co-crystal into co-amorphous mixture by heating. The co-crystal was kept isothermally at 110 °C for 30 min to completely remove the water molecules and the ordered structure collapsed into co-amorphous. Storage of the co-amorphous samples at high relative humidity quickly reverted the samples into co-crystalline, but under dry conditions the samples remained amorphous for 5 months [28].

The improvement of co-amorphous systems has been made by the direct incorporation of components in the form of salt. It was possible to fully prepare co-amorphous sodium naproxen and indomethacin (NSI) systems as well as sodium naproxen, naproxen and indomethacin (NSNI) ternary systems with significantly improved physical stability and increased Tg, up to 40 °C or higher, compared to naproxen-indomethacin (NI) samples. During 270 days of storage, the initial amorphous NSI and NSNI samples did not recrystallize, whereas for the naproxen-indomethacin co-amorphous system recrystallization could not be prevented for more than 112 days irrespective of the preparation method [78]. In addition, for NSI samples, the IDR of naproxen and indomethacin was increased by two-fold compared to NI samples. In conclusion, physical stability and the dissolution rate of the drug in co-amorphous systems can be improved significantly by partial or complete conversion of the drug molecule to its salt analogue, without affecting the pharmacological profile [78].

## 6. Examples of Co-Amorphous Dispersions with Pharmacological Effects

### 6.1. Drug-Drug Combinations

➢ Cimetidine is sometimes co-administered with naproxen for the treatment of NSAID-induced gastro-intestinal disorders. Allesø et al. [17] prepared co-amorphous naproxen-cimetidine SDs with increased Tg by milling. The IDR of naproxen and cimetidine in the co-amorphous SD was respectively four-fold and two-fold higher than the individual crystalline forms. While the dissolution rate of amorphous cimetidine was found to be identical to the crystalline drug due to recrystallization upon contact with the dissolution medium, the dissolution rate of co-amorphous naproxen SD remained high without recrystallization.
➢ RTV ritonavir, a protease inhibitor, was stabilized with indomethacin in co-amorphous SDs, prepared by solvent evaporation. These were stable for 90 days, while the solubility of both drugs was about three-fold greater compared to the corresponding crystalline analogs. Moreover, co-administration of the two drugs improved the efficiency of antiretroviral therapy and the reduction of ritonavir side effects [66].
➢ Co-amorphous naproxen–indomethacin SD was prepared by spray drying [88]. The influence of the process conditions on the resulting initial sample crystallinity and the recrystallization of the drugs was studied. Recrystallization was affected by the total and individual recrystallization rates of the co-amorphous components, the formation of indomethacin polymorphs and by the inlet temperature and feed rate of the spray dryer.
➢ Combined oral therapy of hypoglycemic agents may assist reduction of side effects and costs. Co-amorphous dispersions of nateglinide (NT) and metformin hydrochloride were formed at therapeutic doses and showed a significantly increased release rate of NT [70]. They also showed synergistic action in patients with type 2 diabetes that could not be controlled with monotherapy. Co-administration did not affect the pharmacokinetics.
➢ Recently, an interesting co-amorphous drug-drug system consisting of atenolol (cardio-selective beta blocker) and hydrochlorothiazide (diuretic) was developed by cryomilling for the combined therapy of hypertensive patients. The co-amorphous SD showed better physical stability (30 days) compared to the individual amorphous drugs [38].

### 6.2. Drug-Carboxylic Acids

➢ Masuda et al. [96] studied solid dispersions of acyclovir with different carboxylic acids prepared by cooling or solvent evaporation of drug solutions in various solvents. Acyclovir formed cocrystal with tartaric acid but co-amorphous mixture with citric acid. The amorphized mixture was characterized by XRPD, TG/DSC IR and HPLC.
➢ Ali et al. [97] prepared stable clozapine (CZ) co-amorphous systems with low MW/low Tg carboxylic acids such as citrate, d-tartrate or oxalate, by rapid solvent evaporation in vacuum via formation of H-bonds. The co-amorphous mixture with tartaric acid had the highest dissolution rates (>95% in 20 min) compared to pure crystalline CZ (56%).
➢ In another study by Maher et al. [71], solvent evaporation was also applied to prepare co-amorphous SDs of olanzapine (OL) using polycarboxylic acids as co-formers. The mixtures were incorporated into rapidly dissolving oral polymer films containing additionally HPMC, Na-CMC, glycerin, PG, PEG 400, citric acid, saccharin, menthol as an easy and simple way of administration. Co-amorphization of OL-ascorbic acid was achieved at 1:2 molar ratio through H-bonding and showed a more than 600-fold increase in OL solubility. The films showed high dissolution rate (97% in 10 min). The co-amorphous dispersion in the films remained stable for over 12 weeks at 40 °C/75% RH. Pharmacokinetic data showed improved OL AUC (144 ng h/mL) and Cmax (14.2 ng/mL) compared to commercial formulations.

### 6.3. Drug-Amino Acids

➢ Löbmann et al. [41,42] prepared co-amorphous systems of AAs in combination with carbamazepine (CBZ) and indomethacin (IND) by milling. Selection of AA was based on the binding site at the biological receptors arginine (ARG) and tyrosine (TYR) for IND binding to cyclooxygenase 2, and receptors phenylalanine (PHE), tryptophan (TRP) for CBZ binding to neural Na+ channels. Specific hydrogen bonding and π−π interactions were found in all co-amorphous dispersions that contained at least one binding AA from the biological target site of the drug, whereas the blends of IND with the non-receptor binding AAs PHE and TRP showed no interactions [42]. CBZ could only be prepared as co-amorphous with TRP while IND gave co-amorphous binary mixture with ARG but not with TYR. However, binary co-amorphous IND-amino acid mixtures were also obtained with the non-receptors PHE and TRP. The stability of the co-amorphous SDs was over 6 months, whereas for the pure amorphous it was less than 7 days.
➢ ARG has been found to form co-amorphous salts with weak acids such as NSAIDs and was used successfully as a co-former for indomethacin [6,42]. Additionally, it can make π−π interactions with molecules having aromatic rings, e.g., naproxen-arginine co-amorphous mixture which showed physical stability and 10-fold faster IDR compared to crystalline drug [42].
➢ On the basis of the ability of ARG for co-amorphization it was thought that the structurally similar citrulline (CTL) could also show similar behavior. However, although its incorporation in mixtures with drugs reduced their crystallinity, fully co-amorphous mixture was obtained only with furosemide (FUR-CTL) and nitrofurantoin (NIT-CTL). Hence, although structural similarity is related to molecular mixing, it cannot predict co-amorphization [98].
➢ In the study by Laitinen et al. [74], from the receptor binding aminoacids of simvastatin (SVS): aspartic acid, lysine and serine, the drug formed co-amorphous only with LYS, whereas glibenclamide (GBC) was able to form co-amorphous with both serine (receptor) and threonine (non-receptor) at s 1:1 molar ratio and with glibenclamide-serine-threonine at 1:1:1. Co-amorphous systems showed improved physical stability, for SVC-LYS 3 months and for GBC-SER for 6 months. A possible reason for the stabilization of GBC was prevention by the AA of the conversion of the amide group into the unstable tautomeric imide form.

### 6.4. Co-Amorphous Dispersions with Salt Formation

➢ Valsartan VAL, a selective angiotensin antagonist used in hypertension and heart failure, was processed in a vibrational ball mill with l-histidine (HIS), l-lysine (LYS) and l-Arginine (ARG). Co-amorphous drug-AA binary dispersions were obtained and showed increased solubility and IDR at pH 4.6, 6.8. The increase of solubility achieved from co-amorphous ternary mixtures having a second AA was approximately 1000-fold greater, indicating that the second AA had an important role. The reason for the remarkable IDR increase of VAL from the co-amorphous SD was the ionization and salt drug-amino acid formation, as well as the high-water solubility of AAs. The co-amorphous VAL-ARG and VAL-LYS SDs were stable for 3 months [79].

### 6.5. Examples of Permeability Together with Solubility Improvement

➢ Co-amorphous compositions can address problems of solubility as well as permeability of BCS IV drugs [37]. Apart from the physicochemical properties of the drug molecule (size, polarity), poor permeability may arise if the drugs are substrates to efflux glycoproteins P-gp. These reside in the gastrointestinal epithelium and act as a physiological barrier. In order to overcome these problems, Teja et al. [37] co-amorphisized the poorly water soluble and low permeability drug talinolol, an efflux pump substrate, with naringin, an efflux pump inhibitor. In this way they improved both the dissolution rate and the permeability-absorption of the drug by inhibiting the action of P-gp. The permeability of talinolol was found to increase from 2.48 × 10^−5^ (control value) to 3.16 × 10^−5^ cm/s when formulated as a co-amorphous system. The increase of the bioavailability of talinolol in rats was also improved, while the average AUC was found to be 5.4-fold higher than the crystalline drug, which was attributed to a combination of increased solubility and inhibition of P-gp.

## 7. Formulation of Co-Amorphous Dispersions into Tablets

Since co-amorphous systems are not in dosage form themselves, they have to be formulated into a final product.

**Figure 13:**
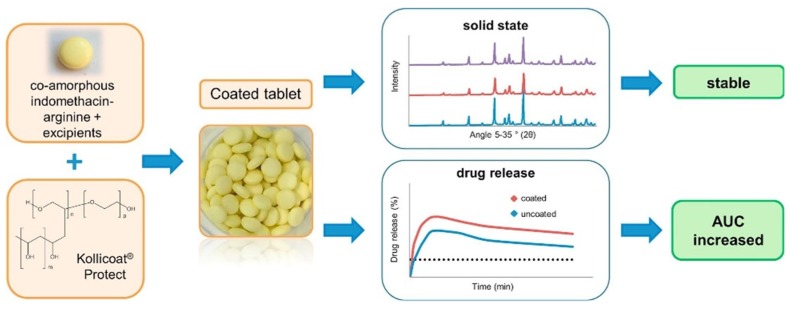
Preparation of stable polymer-coated tablets of co-amorphous materials [101].

➢ Processing into a tablet or capsules form has the risk of crystallization due to the influence of environmental factors (moisture, temperature), while mechanical stresses during compression into tablets may alter the amorphous structure, the stability and the dissolution profile [18,23,99]. Other problems arising from the soft and sticky nature of the amorphous SDs may be difficulty in granulation and sieving, poor flowability and compressibility [100]. Wet granulation is not recommended since the added water may cause plasticization and crystallization. Also, during melt granulation, the heat input could increase the temperature above the Tg of the co-amorphous components with the risk of recrystallization.
➢ Lenz et al. [18] were the first to report a successful tablet formulation of the co-amorphous indomethacin-arginine (IND-ARG) dispersion prepared by spray drying and compressed into tablets using mannitol, croscarmellose sodium, colloidal silicon dioxide, magnesium stearate as tableting aids. Molecular interactions were unaffected by compression and no crystallization occurred. Tablets showed immediate release of IND, four-fold supersaturation concentration over crystalline IND and long-term stability. AUC was not affected by tableting compared to spray dried co-amorphous IND-ARG powder. The AUC at 24 h of the IND-ARG tablets was two-fold that of the tablets of physical mixtures of the crystalline drugs (IND-ARG) and three-fold that of the IND. The dissolution plateaus after 24 h were different for spray dried IND-ARG tablets, physical mixtures IND-ARG tablets and neat spray dried IND-ARG powder which was explained due to the formation of different indomethacin polymorphs.
➢ Long-term stability of co-amorphous systems can be ensured by the application of polymeric coatings. Petry et al. [101] prepared a co-amorphous indomethacin-arginine homogeneous system (SD IND-ARG) by spray drying, which was then processed into tablets and coated with polyvinyl alcohol-polyethylene glycol graft copolymer (Kollicoat^®^ Protect). The steps of the processing are shown in Figure 13. Contact with water, heat and stress during coating did not induce recrystallization of IND-ARG. The coated tablets were stable for at least 94 days, and there was no recrystallization after storage at RT/75% for 91 days, which demonstrated high physical stability even under humid conditions. They also found that the applied coating enhanced dissolution of the co-amorphous tablets by about 30% compared to neat spray dried IND-ARG.

## 8. Current Issues, Limitations and Potential Solutions

Co-amorphous solid dispersions appear as a very challenging approach for the formulation of drugs in amorphous state, having the benefits of improved solubility and absorption but also of the lower amounts of required excipients, allowing room for high dosing. More conventional combinations of drugs with carboxylic acids and modern combinations of drugs with receptor-binding amino acids can be used as a platform for the selection of suitable co-amorphous systems. The concept of the receptor binding biomolecules to various drugs can be further exploited. Emphasis should be placed on the development of potential products based on co-amorphous dispersions using methods such as milling, spray drying and hot melt extrusion that can be realized on a large industrial scale. Processing conditions should be carefully selected to obtain stable co-amorphous formulations and single or combined analytical techniques should be applied in the context of Process Analytical Technology to monitor the molecular state and interactions during processing.

The considerable number of over 20 commercial products of single amorphous dispersions [102] gives good grounds for the possibility of commercializing co-amorphous dispersions. However, transformation during storage to crystalline phases is a possibility resulting in failure of dissolution rate specification and has been the reason that products of single amorphous drugs have been recalled by the FDA. Therefore, although as already described in this review, physicochemical evaluation and stability tests showed that the amorphous state and mixture homogeneity of the co-amorphous SDs can be retained for about 1-year, further data over longer periods of time are required to ensure commercial viability. Newer analytical techniques applied in conjunction [68,73] could help immensely in the evaluation of the molecular state and detect kinetically controlled possible interactions between the co-amorphous SD components, providing more insights into the mechanisms of phase separation and crystallization from the early processing stages to long storage periods.
